# Assessment of Cytotoxic Activity of Rosemary (*Rosmarinus officinalis* L.), Turmeric (*Curcuma longa* L.), and Ginger (*Zingiber officinale* R.) Essential Oils in Cervical Cancer Cells (HeLa)

**DOI:** 10.1155/2016/9273078

**Published:** 2016-11-30

**Authors:** P. A. S. R. Santos, G. B. Avanço, S. B. Nerilo, R. I. A. Marcelino, V. Janeiro, M. C. Valadares, Miguel Machinski

**Affiliations:** ^1^Laboratory of Toxicology, Department of Basic Health Sciences, State University of Maringá, Maringá, PR, Brazil; ^2^University Center Inga (UNINGÁ), Maringá, PR, Brazil; ^3^Laboratory of Pharmacology and Cellular Toxicology-LFTC/FARMATEC, School of Pharmacy, Federal University of Goiás, Goiania, GO, Brazil; ^4^Department of Statistics, State University of Maringá, Maringá, PR, Brazil

## Abstract

The objective of this study was to evaluate the cytotoxic activity of rosemary (REO,* Rosmarinus officinalis* L.), turmeric (CEO,* Curcuma longa* L.), and ginger (GEO,* Zingiber officinale* R.) essential oils in HeLa cells. Cytotoxicity tests were performed* in vitro*, using tetrazolium (MTT) and neutral red assays for evaluation of antiproliferative activity by different mechanisms, trypan blue assay to assess cell viability and evaluation of cell morphology for Giemsa to observe the cell damage, and Annexin V to evaluate cell death by apoptosis. CEO and GEO exhibited potent cytotoxic activity against HeLa cells. IC_50_ obtained was 36.6 *μ*g/mL for CEO and 129.9 *μ*g/mL for GEO. The morphology of HeLa cells showed condensation of chromatin, loss of cell membrane integrity with protrusions (blebs), and cell content leakage for cells treated with CEO and GEO, from the lowest concentrations studied, 32.81 *μ*g/mL of CEO and 32.12 *μ*g/mL of GEO. The Annexin V assay revealed a profile of cell death by apoptosis for both CEO and GEO. The results indicate cytotoxic activity* in vitro* for CEO and GEO, suggesting potential use as anticancer agents for cervical cancer cells.

## 1. Introduction

The use of essential oils (EOs) of aromatic plants for dietary and therapeutic purposes has been a focus on research in health sciences due to their different biological properties such as antimicrobial, analgesic, anti-inflammatory, antiparasitic, antioxidant, and antitumoral effects [1–4]. The main phytochemicals found in EOs are mono and sesquiterpenes, which confer organoleptic characteristics to the EOs as well as their biological activities [5, 6]. In addition, these secondary metabolites have shown low side effects and toxicity [6].

The dried leaves of rosemary (*Rosmarinus officinali*s L.), Lamiaceae family, are used as a food condiment and to enhance or change the flavor of foods [7]. Turmeric (*Curcuma longa* L.) and ginger (*Zingiber officinale* R.) are members of the Zingiberaceae family. Turmeric is a native plant from India and South Asia. However, it has now been found worldwide and widely used as a spice, giving foods a characteristic flavor and color. Ginger originates from Southeast Asia and has marked characteristics of odor and hot flavor [8].

Cancer is a public health problem particularly in developing countries. In these countries, it is estimated that the impact of cancer in the population corresponds to approximately 80% from the 20 million new cases estimated for 2025 [9]. In Brazil, National Institute of Cancer (INCA) estimates for the 2016-2017 period around 600,000 new cases of cancer. A total of 16,340 new cases of cervical cancer are expected in 2016, with an estimated risk of 15.85 cases per 100,000 women [9]. In Latin America, cervical cancer significantly contributes to incidence of cancer among women, being the second cause of death in women.

The antitumor property of EOs has been the source of investigation for the development of drugs to treat different types of cancer. There is the perspective that EOs can be used as a therapeutic agent and confer benefits for human health, provided their toxicity can be established. The objective of this study was to evaluate the cytotoxic activity of rosemary (*R. officinali*s, REO), turmeric (*C. longa*, CEO), and ginger (*Z. officinale*, GEO) essential oils in HeLa human cervical cancer cells, aiming at its determining potential anticancer activity.

## 2. Material and Methods

### 2.1. Extraction and Characterization of EOs

Rosemary (REO), turmeric (CEO), and ginger (GEO) essential oils were previously obtained by hydrodistillation of dried leaves of* R. officinalis* [10] and rhizomes of* C. longa* [11] and* Z. officinale* [12], according to the European Pharmacopoeia [13]. The EOs were stored at 4°C in flasks shielded from light until time of use. Identification of the principal components was performed by chromatography in gaseous phase coupled to a mass spectrometer (CG-MS) and by nuclear magnetic resonance (NMR) spectroscopy.

### 2.2. Cell Cultures

HeLa human cervical cancer and HepG2 human liver cells were obtained from the Rio de Janeiro Cell Bank (Rio de Janeiro, RJ, Brazil). Each cell line was cultured in complete Dulbecco's Modified Eagle's Medium (DMEM, Sigma-Aldrich, St. Louis, MO, USA), supplemented with 20% (v/v) fetal bovine serum (FBS) (GIBCO-Invitrogen, Indianapolis, IN, USA), solution containing penicillin (100 IU/mL) and streptomycin (100 *μ*g/mL) (Sigma-Aldrich, St. Louis, MO, USA), and 2 *μ*g/mL amphotericin B (Sigma-Aldrich, St. Louis, MO, USA). The cells were kept in an incubator (Panasonic®, Chicago, IL, USA) in a humidified atmosphere of 5% CO_2_ in air at 37°C [14, 15].

### 2.3. 3-[4,5-Dimethylthiazol-2-yl]-2,5-diphenyltetrazolium Bromide (MTT) Assay

MTT reduction test, adapted from Mosmann [16], was carried out to evaluate cell viability via mitochondrial toxicity of the EOs. Briefly, the cells (1 × 10^6^ cells/mL) were seeded onto 96-well plates overnight. After that, the cells were treated with complete medium only (control cells) or seven different concentrations of REO (31.12–1192 *μ*g/mL), CEO (32.81–2100 *μ*g/mL), or GEO (20.12–1928 *μ*g/mL) for 24 h. Posteriorly, the cells were washed with PBS and 100 *μ*L of medium (DMEM + 2% SFB) containing MTT (0.5 mg/mL) (Sigma-Aldrich, St. Louis, MO, USA) was added in each well. The microplate was incubated for 3 h. After this period the solution was discarded by inversion and 100 *μ*L of dimethyl sulfoxide (DMSO) (Sigma-Aldrich, St. Louis, MO, USA) was added. The plate was placed in a microplate shaker at 250 rpm for 15 minutes. Absorbance was determined at 540 nm in a spectrophotometer (Bio Tek Power Wave XS,Winooski-Chittenden, VT, USA). This assay was performed in triplicate.

### 2.4. Neutral Red Uptake (NRU) Assay

NRU assay [17] was used to evaluate the cell viability via lysosomal toxicity of EOs. The cells (1 × 10^6^ cells/mL) were seeded onto 96-well plates overnight. Then, the cells were treated with complete medium only (control cells), REO, CEO, and GEO, at the same concentrations used for the MTT assay, for 24 h. After that, the supernatant of each well was removed and 100 *μ*L of DMEM containing 5% (v/v) FBS and neutral red (NR, 0.25 mg/mL) was added. After 3 h of incubation, NR medium was discarded and the cells were washed with PBS. Posteriorly, 100 *μ*L of NR desorb solution (50 EtOH : 1 acetic acid : 49 water) was added to the wells. The plates were shaken and the absorbance was measured at 550 nm.

### 2.5. Trypan Blue Assay

Trypan blue dye is used to analyze cell viability via membrane rupture, since it does not cross intact membrane of viable cells. Thus, stained cells are scored as dead. Briefly, the cells (1 × 10^6^ cells/mL) were seeded onto plate overnight. Then, the cells were treated with complete medium only (control cells), REO, CEO, or GEO, at five different concentrations (250–2000 *μ*g/mL) for 24 h. After that, the cells were removed from the plate and an aliquot of cell suspension was diluted with trypan blue solution (1 : 10) (Sigma-Aldrich, St. Louis, MO, USA). The cells were then observed under light microscopy (Nikon T1-SM, Eclipse, Konan, Tokyo, Japan) and the viability of the cells was estimated using a Neubauer Chamber.

### 2.6. Cellular Morphology Evaluation

Cell death can be characterized by several morphological changes such as cellular retraction, loss of adhesion, and chromatin condensation. The cell membrane can form extensions (blebs) and then the nucleus disintegrates into fragments surrounded by nuclear membrane, originating apoptotic bodies [18]. Thus, morphology evaluation of cells treated with EOs was carried out by Giemsa staining based on the protocol described by Mota et al. [19]. Briefly, the cells were overnight seeded onto 24-well plates containing a coverslip in each well. Then, the cells were treated with complete medium only (control cells), REO, CEO, or GEO, at the same concentrations used for the MTT assay, for 24 h. After that, the coverslips were removed and washed in PBS, fixed in methanol, and left at room temperature for 20 minutes. Posteriorly, the cells were stained for 10 minutes followed by washing in water. The slides were analyzed by a light microscope (Olympus BX41 microscope, Cybernetics, Silver Spring, MD, USA), using a 40x objective, and photographed using a high resolution camera (Olympus American INC).

### 2.7. Apoptosis Evaluation by an Annexin V Binding Assay

Annexin V binding assay allows identifying changes in the plasma membrane, which occur during the early events of the cell death process by apoptosis [20]. The assay was carried out using an Alexa Fluor® 488/Dead Cell Apoptosis Kit (Life Technologies, Waltham, MA, USA), according to the manufacturer's instructions. In brief, the cells (1 × 10^6^ cells/mL) were seeded onto 24-well plate overnight. The cells were then treated with complete medium (control cells), camptothecin (positive control), REO, CEO, or GEO for 24 h. After that, the supernatant of each well was removed and the cells were washed with PBS. Then, 400 *μ*L of Annexin-binding buffer (10 mM HEPES, 140 mM NaCl, and 2.5 mM CaCl_2_, pH 7.4) and 10 *μ*L of Annexin V were added in each well. The plates were incubated at room temperature, protected from light, for 15 min. After incubation, the cells were analyzed by a fluorescence microscopy (EVOS FL Cell Imaging System, Thermo Fisher Scientific, Waltham, MA, USA).

### 2.8. Statistical Analysis

Different statistical treatments were applied for the cytotoxicity assays. For the trypan blue assay, application of Kruskal-Wallis rank sum tests was necessary. In cases where the test detected significant difference at up to 10% significance, the Bonferroni pairwise comparison test was applied. The Quadratic Logistic Regression Model was applied for the other assays using the estimated parameters on the Quadratic Logistic Regression Model. The results were analyzed on the R software program version 3.2.1 (Team R Development Core 2015) using the “RDC” package [21].

(1) $$\begin{array}{c} \text{Model: }f\left(\;dose\;\right)=c+\frac{d-c}{1+e^{b\left(\log\left(dose\right)-e\right)}}. \end{array}$$

## 3. Results and Discussion

### 3.1. Chemical Composition of the EOs

The chemical characteristics of REO, CEO, and GEO are demonstrated in Table 1. The bioactive compounds of the EOs are responsible for conferring the biological properties of each EO, thereby characterizing each EO in terms of benefit or risk. These characteristics, besides interacting with one another [22], may also be influenced by numerous factors, such as the geographic location of planting and the method by which the EO is extracted [23].

### 3.2. Assessment of Cytotoxicity

**Figure 1:**
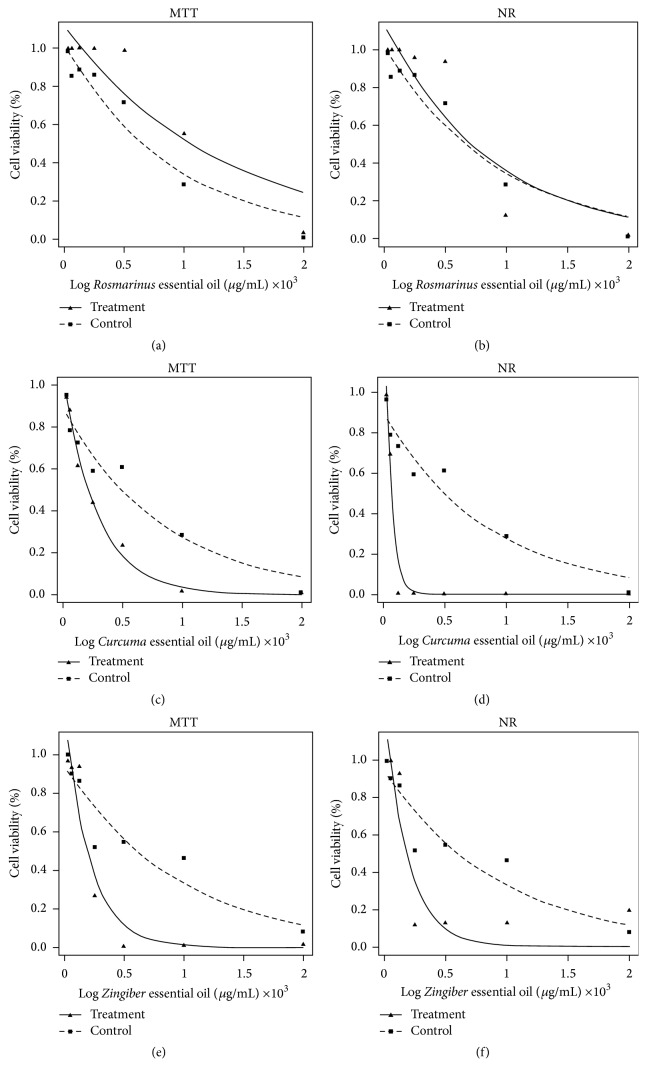
Comparison of cytotoxic effect of* R. officinalis* (REO),* C. longa* (CEO), and* Z. officinale* (GEO) essential oils in cell lines HeLa (treatment) and HepG2 (control) on MTT and NR assays. (a, b) IC_50_ result for REO on MTT and NR. (c, d) IC_50_ results obtained for CEO on MTT and NR. (e, f) IC_50_ results obtained for GEO on MTT and NR. Cell density was 1 × 10^6^ cells/mL (*n* = 3).

The REO, CEO, and GEO were submitted to MTT and NR assays to assess cytotoxicity activity (Table 2, Figure 1). The results showed that both CEO and GEO exhibited cytotoxic activity against the cervical cancer cells; that is, both of these EOs reduced cell viability. GEO showed cytotoxic activity on both tests (MTT/NR), whereas CEO exhibited greater cytotoxicity on the NR test (Figure 1(d)). Reduced cell viability at the lowest concentration can be observed compared to the other EOs tested.

These results indicate that REO exhibited no significant cytotoxic activity in HeLa cells (treatment) in comparison to HepG2 cells (control) (Figures 1(a) and 1(b)). The major components of REO were monoterpenes (1.8-cineole and *α*-pinene), while main components for CEO and GEO were sesquiterpenes and oxygenated monoterpenes. Both CEO and GEO showed significant cytotoxic activity in HeLa cells in comparison to HepG2 cells (Figures 1(d), 1(e), and 1(f)). Wang et al. [24] found that the compound 1.8-cineol had low toxicity in tumor lines, a result corroborated by our finding for REO. According to Srivastava et al. [25], monoterpene compounds are more active against cancer cells than monoterpene or sesquiterpene compounds.

The studies conducted by Tyagi et al. [26] evaluate the anticancer potential of nine compounds from CEO. The authors showed that only the compound *β*-sesquiphellandrene had strong antiproliferative activity in different tumor cells and also exhibited synergism with chemotherapeutic agents. In the present study, the compound *β*-sesquiphellandrene was the fifth-most prevalent compound in CEO (2.4%), where this may also have an effect on the suppression of cervical cancer colonies in combinatorial action with other compounds identified.

### 3.3. Analysis of Antiproliferative Activity by Trypan Blue Exclusion

The concentrations of REO, CEO, and GEO used in this experiment in HeLa cells (250; 500; 1,000; 1,500; and 2,000 *μ*g/mL) showed no significant difference in comparison to HepG2 cells (control). However, comparison of concentrations pairwise yielded significant differences (*p* value 1.0). The significant comparisons between pairs were 2,000-1,500 (*p* = 0.5178) and 2,000-250 (*p* = 0.7496) for CEO; 2,000-1,000 (*p* = 0.9089) and 2,000-250 (*p* = 0.0423) for REO; and 2,000-250 (*p* = 0.036) and 1,500-250 (*p* = 0.4382) for GEO. Based on these results, it can be noted that GEO had greater antiproliferative activity against HeLa cervical cancer cells at a lower concentration. Our results corroborate those of the studies by Jeena et al. [27] assessing the cytotoxic and antitumor activity of GEO in Dalton's lymphoma ascites (DLA) tumor cell line. The study results showed that GEO exhibited potent cytotoxic and antiproliferative activity* in vitro* in DLA (L929) cells. The authors also highlighted the idea that GEO showed antitumor potential and may be used as an anticancer agent.

### 3.4. Morphological Analysis

**Figure 2:**
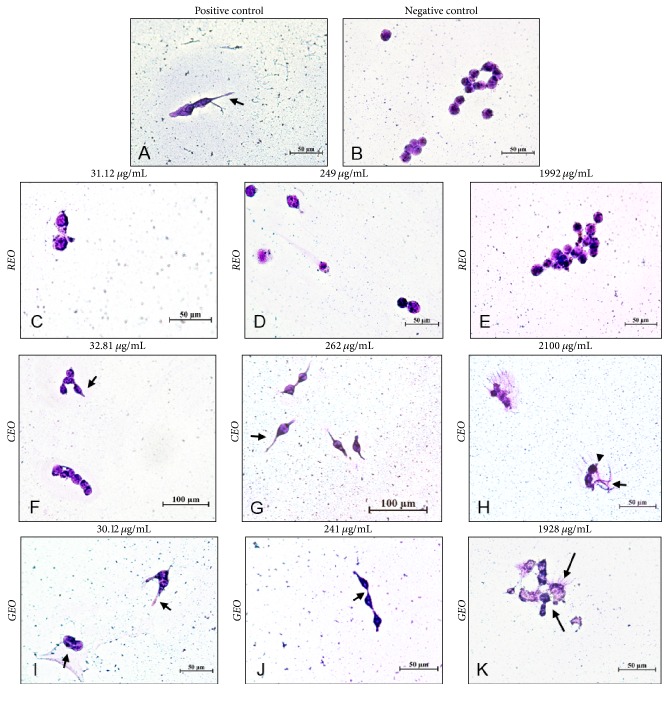
Morphological changes in HeLa cells treated with* R. officinalis* (REO),* C. longa* (CEO), and* Z. officinale* (GEO) essential oils and dyed with Giemsa. (A) Positive control: cell population treated with DMSO; (B) negative control: cell population without influence of treatment; (C–E) cells treated with 31.12; 249; and 1992 *μ*g/mL of REO; (F–H) cells treated with 32.81; 262; and 2100 *μ*g/mL of CEO; (I–K) cells treated with 30.12; 241; and 1928 *μ*g/mL of GEO. Cell density 1 × 10^6^ cells/mL. Images taken at 40x magnification. (→ cell membrane protrusions (“blebs”); ▸ chromatin condensation; ⟶ cell content leakage and formation of apoptotic bodies.)

For the EOs to exert such cytotoxic and antiproliferative activity, several different mechanisms may be involved. Possible mechanisms include induction of cell death by apoptosis and/or necrosis, arrest of the cell cycle, and loss of function of key cell organelles [28]. In the present study, cell morphology of HeLa after REO, CEO, and GEO exposure was analyzed. The cells demonstrated in Figures 2(C), 2(D), 2(E) and 2(F) have intact organelles, organized cytoplasm, and complete cell membrane similar cells of negative control (NC). REO did not demonstrate cytotoxic effect in HeLa cells. However, cell membrane protrusions called “blebs” (Figure 2(G)) and cell content leakage (Figure 2(H)) were observed in the CEO at 262 and 2100 *μ*g/mL, respectively. For GEO, cell membrane protrusions were visualized in 30.12 *μ*g/mL (Figure 2(I)); blebbing and chromatin condensation occurred at 241 *μ*g/mL (Figure 2(J)). GEO at 1928 *μ*g/mL (Figure 2(K)) presented amorphous cells, blebs, cytoplasm leakage, and formation of apoptotic bodies, containing nuclear fragments or otherwise, which can be visualized.

**Figure 3:**
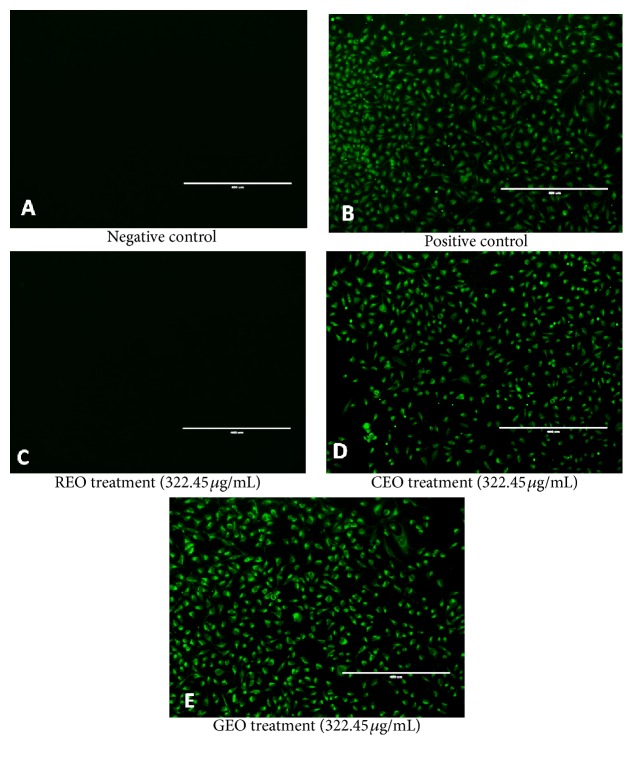
Analysis of apoptotic morphology of HeLa cells treated with* R. officinalis* (REO),* C. longa* (CEO), and* Z. officinale* (GEO) essential oils using the Annexin V assay. (A) Negative control: cell population without influence of treatment. (B) Positive control: cell population treated with camptothecin. (C, D, and E) Cell population exposed to treatment with 322.45 *μ*g/mL of REO, CEO, and GEO. Analysis performed by fluorescence microscopy. Images taken at 10x magnification.

Our results also show the same characteristic morphological patterns of cell death by apoptosis for CEO and GEO, causing HeLa cells to exhibit changes in their morphology at the lowest concentration studied (Figures 2(F)–2(K)). CEO and GEO exhibited strong cytotoxic activity against cervical cancer cells. The Annexin V assay was performed to confirm cell death by apoptosis (Figure 3). Exposure of HeLa cells to REO, CEO, and GEO at the concentration of 322.45 *μ*g/mL revealed that REO caused no cell death by apoptosis. CEO and GEO, however, showed positive results similar to those of the positive control (camptothecin).

The bioactive properties of EOs have attracted growing interest from scholars seeking to unveil the mechanisms of action of these natural products, prompting an increase in studies assessing the antiproliferative, antitumor, and anticancer activity of these compounds [29].

## 4. Conclusion

Oxygenated monoterpene compounds present in turmeric (*C. longa*) and ginger (*Z. officinale*) essential oils were possibly responsible for presenting better antitumor activity. CEO and GEO showed effective cytotoxic activity against human cervical cancer cells (HeLa), inducing significant reduction in cell viability of these tumor cells. Our results clearly show that this cytotoxicity was responsible for inducing cellular death in human cervical cancer cell by apoptosis. Therefore, both CEO and GEO can be considered promising chemotherapeutic agents in the treatment of cervical cancer. Further in-depth studies determining the mechanism of action of both CEO and GEO, along with their components, must be warranted.

## Figures and Tables

**Table 1:** Components of essential oils of dried leaves of *R. officinalis* (REO) and rhizomes of *C. longa* (CEO) and *Z. officinale* (GEO) from Southern Brazil, identified by GC/MS.

Components	Percentage (%)		
	REO	CEO	GEO
*Monoterpenes*			
*α*-Pinene	12.4^*∗*^	0.6	6.0
Camphene	3.7	—	16.4^*∗*^
*β*-Pinene	1.8	—	0.7
p-Cymene	2.1	0.8	0.1
*β*-Myrcene	0.7	—	—
1.8-Cineole	52.2^*∗*^	0.7	8.9^*∗*^
3-Carene	0.2	—	—
Myrcene	—	—	1.9
*α*-Terpinene	0.4	—	
*trans*-*β*-Ocimene	0.1	—	—
*γ*-Terpinene	0.4	—	—
*cis*-Ocimene	—	—	0.2
Terpinolene	—	—	0.4
6-Camphenol	0.1	—	—
*Oxygenated monoterpenes*			
Vinyl propionate	—	1.7	—
Tricyclene	—	—	0.4
Sabinene	—	—	0.1
Camphor	15.2^*∗*^	0.1	—
*α*-Phellandrene	0.1	—	0.7
*β*-Phellandrene	—	—	8.8^*∗*^
*α*-Terpineol	2.3	0.2	0.6
4-Terpineol	0.5	—	—
*γ*-Curcumene	—	0.5	—
*α*-Turmerone	—	23.5^*∗*^	—
*β*-Turmerone	—	22.7^*∗*^	—
Limonene	3.5	—	—
Linalool	0.4	—	0.6
Borneol	3.0	—	0.9
Isoborneol	0.1	—	—
Citronellol	—	—	0.5
Neral	—	—	4.6
Geraniol	—	—	2.4
*Sesquiterpenes*			
*β*-Sesquiphellandrene	—	2.4	—
*β*-Caryophyllene	—	0.4	—
ar-Turmerol	—	1.5	—
ar-Turmerone	—	33.2^*∗*^	—
ar-Curcumene	—	2.6	—
*α*-Cadinol	—	1.3	—
(*6R*,*7R*)-Bisabolone	—	3.1	—
(E)-*α*-Atlantone	—	1.4	—
geranial	—	—	9.9^*∗*^
*α*-zingiberene	—	1.0	—

Source: Bomfim et al. [10], Ferreira et al. [11] and Nerilo et al. [12]. ^*∗*^Majority components of essential oils.

**Table 2:** IC_50_ values obtained by cytotoxicity assays (MTT: *3-(4,5-dimethylthiazol-2-yl)-2,5-diphenyltetrazolium bromide* and NR: *3-amino-7-dimethylamino-2-methylphenazine*) in HeLa (treatment, T) and HepG2 (control, C) cells treated with *R. officinalis* (REO), *C. longa* (CEO), and *Z. officinale* (GEO) essential oils.

Essential oil	MTT-IC_50_ (*μ*g/mL)		IC_50C_/IC_50T_	*p* value
	C (HepG2)	T (HeLa)		
REO	633.0	909.6	0.696	0.1502
CEO	614.7	211.6	3.051	0.1984
GEO	635.1	141.4	4.330	0.0549^*∗∗*^

	NRU-IC_50_ (*μ*g/mL)		IC_50C_/IC_50T_	*p*-value
	C (HepG2)	T (HeLa)		

REO	633.0	909.6	0.696	0.1502
CEO	614.7	36.6	16.795	0.0169^*∗*^
GEO	635.1	129.9	4.489	0.0632^*∗∗*^

^*∗*^Significant difference at 5%.

^*∗∗*^Significant difference at 10%.
